# Updates on protein-prenylation and associated inherited retinopathies

**DOI:** 10.3389/fopht.2024.1410874

**Published:** 2024-07-04

**Authors:** Sudhat Ashok, Sriganesh Ramachandra Rao

**Affiliations:** ^1^ Department of Ophthalmology, Jacobs School of Medicine and Biomedical Sciences, State University of New York, University at Buffalo, Buffalo, NY, United States; ^2^ Neuroscience Program, Jacobs School of Medicine and Biomedical Sciences, State University of New York, University at Buffalo, Buffalo, NY, United States; ^3^ Research Service, VA Western New York Healthcare System, Buffalo, NY, United States

**Keywords:** post-translational modifications, mevalonate pathway, retina, prenylation, farnesylation, geranylgeranylation, inherited retinopathies

## Abstract

Membrane-anchored proteins play critical roles in cell signaling, cellular architecture, and membrane biology. Hydrophilic proteins are post-translationally modified by a diverse range of lipid molecules such as phospholipids, glycosylphosphatidylinositol, and isoprenes, which allows their partition and anchorage to the cell membrane. In this review article, we discuss the biochemical basis of isoprenoid synthesis, the mechanisms of isoprene conjugation to proteins, and the functions of prenylated proteins in the neural retina. Recent discovery of novel prenyltransferases, prenylated protein chaperones, non-canonical prenylation-target motifs, and reversible prenylation is expected to increase the number of inherited systemic and blinding diseases with aberrant protein prenylation. Recent important investigations have also demonstrated the role of several unexpected regulators (such as protein charge, sequence/protein-chaperone interaction, light exposure history) in the photoreceptor trafficking of prenylated proteins. Technical advances in the investigation of the prenylated proteome and its application in vision research are discussed. Clinical updates and technical insights into known and putative prenylation-associated retinopathies are provided herein. Characterization of non-canonical prenylation mechanisms in the retina and retina-specific prenylated proteome is fundamental to the understanding of the pathogenesis of protein prenylation-associated inherited blinding disorders.

## 1 Introduction

Post-translational modifications (PTMs) of proteins are covalent chemical modifications of target peptide motifs which influences protein folding, structure, stability, localization, protein-protein interactions, and function (1, 2). Protein lipidation is a class of PTMs involving enzymatic conjugation with various lipid molecules such as cholesterol (3), phospholipids (4), glycosylphophatidylionositine (GPI), and isoprenes (5). Protein lipidation facilitates protein anchorage to the plasma membrane, protein-protein interactions, and membrane-cytoskeleton interaction (6, 7). The focus of this review article includes isoprene-modification of proteins, and inherited retinal dystrophies with underlying protein prenylation defects.

Protein prenylation involves thioether linkage of an isoprenoid molecule, either a 15-carbon farnesyl pyrophosphate (FPP) or a 20-carbon geranylgeranyl pyrophosphate (GGPP), to one or more cysteine residues of a carboxy terminal target peptide motif (8–10). The biological role of prenylation is best known in the context of cancer biology. Ras GTPases, well-known prenylated protein targets, play critical roles in signal transduction, cell growth, and are implicated in various forms of cancer and cancer progression. For review of Ras prenylation, the reader is referred to the following review articles (11, 12).

In this review, we aim to provide a thorough background of protein prenylation mechanisms, recent advances in approaches to study protein prenylation, and to discuss the role of prenylated proteins in retinal physiology and pathology. We also discuss putative approaches for determining prenylated proteome in the retina.

### 1.1 Biochemical basis of protein prenylation: *de novo* synthesis of isoprenes, and signal sequence-dependent enzymatic prenylation

**Figure 1:**
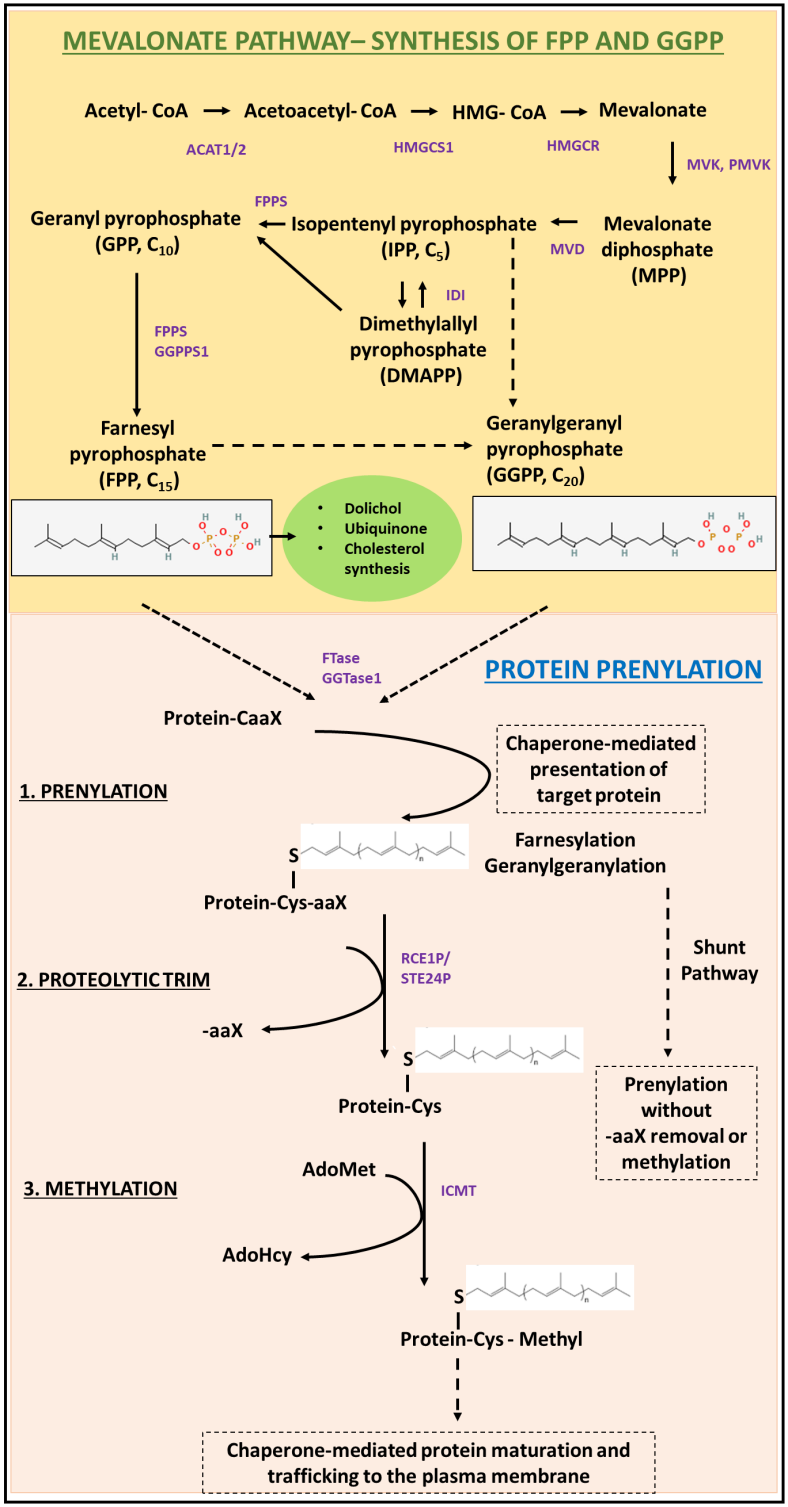
Schematic representation of the biochemical requirements and processes involved in protein prenylation. The mevalonate/cholesterol synthesis pathway is required for the generation of the isoprene moieties (FPP and GGPP) required for protein prenylation. A detailed description of the pre-squalene pathway essential for *de novo* synthesis of isoprenes has been described in Section 1.1. The prenyl groups are then conjugated to target motifs by the action of prenyltransferases (FTase or GGTase). Following prenylation, the target protein undergoes sequential proteolysis (by RCE1), and subsequently methylated before protein maturation and chaperone-mediated membrane trafficking. Alternatively, some prenylated proteins remain cytosolic, through the shunt pathway; without further trafficking to the plasma membrane.

The lipid moieties required for protein prenylation, FPP and GGPP, are key isoprenoid intermediates of the mevalonate/cholesterol synthesis pathway (**Figure 1**). Briefly, acetyl-CoA is converted to HMG-CoA in a sequential manner catalyzed by ACAT1, ACAT2, and HMG-CoA synthase 1 (HMGCS1), which is then converted to mevalonate by HMG-CoA reductase (HMGCR) (13). HMGCR is the rate-limiting step in the mevalonate pathway and is the mechanistic target of statins, a commonly used drug used in the treatment of hypertension and hypercholesteremia (14). Mevalonate kinase (MVK) catalyzes the phosphorylation of mevalonic acid to phosphomevalonate and is subject to feedback inhibition by downstream isoprene intermediates like geranylpyrophosphate (GPP), FPP, and GGPP (15). Phosphomevalonate is converted to isopentenyl pyrophosphate (IPP) by phosphorylation and decarboxylation, catalyzed by phosphomevalonate kinase (PMK) and mevalonate pyrophosphate decarboxylase (MPDC), respectively (16). IPP undergoes reversible isomerization to dimethylallyl pyrophosphate (DMAPP). The condensation of IPP and DMAPP results in the 10-carbon isoprenoid geranyl pyrophosphate (GPP). Subsequent condensation of 1 or 2 more IPP units to GPP yields the 15-carbon and the 20-carbon isoprenoids FPP and GGPP, respectively (17). Key mevalonate pathway intermediates IPP, FPP, and GGPP feed into the synthesis of squalene, dolichols, ubiquinone, sterols and derivative steroid hormones, as well as protein prenylation (18). A schematic representation of isoprene synthesis through the mevalonate pathway is provided in **Figure 1**.

After isoprenoid generation, irreversible prenylation is catalyzed by protein farnesyltransferase (FTase) or protein geranylgeranyltransferase type I (GGTase-I), which differ in target carboxy-terminal signal peptide recognition and the isoprenoid substrate (9). FTase catalyzes the addition of farnesyl pyrophosphate (FPP), while GGTase-I catalyzes the addition of geranylgeranyl pyrophosphate (GGPP, **Figure 1**) (8–10, 19, 20). The C-terminal recognition motif for prenylation is the CAAX (also referred to as Ca_1_a_2_X) domain, in which ‘C’ is the cysteine to be prenylated, ‘A’ is any aliphatic amino acid, and ‘X’ is an amino acid which determines enzyme specificity (8, 21, 22). The target motifs for FTase typically have serine, methionine, alanine, or glutamine at the X residue position, while GGTase-I usually recognizes leucine or phenylalanine. Some substrates may also be recognized by both enzymes as targets for prenylation (23–26). Recent studies have shown that proteins terminating in C(X)_3_X and CXX may also potentially be substrates of FTase, where X represents any amino acid (27–29).

Following prenylation at the cysteine residue in the target motif, most prenylated proteins then undergo two additional modifications before transport to the cell membrane. First, the terminal -AAX residues are cleaved by the CAAX proteases Ras converting enzyme 1 (RCE1P) or zinc metallopeptidase (STE24). The resulting carboxylate then undergoes methylation by the S-adenosylmethionine-dependent isoprenylcysteine methyltransferase (ICMT) to form a post-translationally modified prenylcysteine methyl ester C-terminus on the protein (30, 31) (**Figure 1**). The three steps (prenylation, proteolytic cleavage, and methylation) described above are necessary for function of most prenylated proteins but recently it has been found that heat shock protein chaperone Ydj1p in yeast undergoes prenylation without subsequent proteolysis or methylation in what has been deemed the “shunt pathway”, with evidence of these processing steps actually being deleterious to the protein’s function (32).

The other enzyme responsible for the transfer of 20-carbon geranylgeranyl-group from GGPP to the target motif is termed geranylgeranyltransferase type II (GGTase-II). GGTase-II is unique in that it can modify diverse C-terminal motifs from the Rab family of proteins including CCXX, CXC, or XXCC, where cysteine residues undergo geranylgeranylation and X represents any amino acid (33–36). GGTase-II also requires Rab escort protein (REP) for substrate recognition. REP binds to the substrate motif to be prenylated and presents the catalytic site of GGTase-II for prenylation (37). Recently, a fourth non-canonical prenyltransferase, GGTase-III, was identified which adds a second isoprenoid geranylgeranyl group onto a pre-farnesylated protein substrate with a -CC_Far_AIM C-terminal motif (38, 39). Interestingly, a chemical proteomic study described the discovery of non-canonical prenylated protein, ALDH9A1, which lacks any classic prenylation motif (40). Moreover, this modification was found to be reversible and not inhibited by known prenyltransferase inhibitors suggesting the possibility of a yet-to-be identified prenyltransferase (40). Single cell RNA seq data analysis of the developing mouse retina (41, 42) suggests the expression of this non-canonical prenylated protein in majority of cell types in the developing retina. Taken together, the discoveries of non-canonical C-terminal sequences for FTase recognition, GGTase-III, and the possibility of other non-canonical prenyltransferases have challenged our understanding of this modification and have greatly expanded the potential pool of prenylated proteins.

### 1.2 The molecular basis of prenyltransferase specificity

The mechanistic understanding of the isoprene substrate (FPP vs. GGPP) specificity of prenyltransferases stems from structural studies, structure-function studies, peptide library studies, computational approaches, radiolabeling, and affinity tagging approaches (22, 43–45). Both FTase and GGTase-I are heterodimeric metalloenzymes consisting of an identical α subunit and distinct, homologous β subunits (46–48). The isoprene-binding site of both enzymes is constituted by the interface of the α and β subunits, and predominantly consists of amino acid residues of the β subunit. The isoprene binding pocket of both GGTase-I and FTase is lined with conserved aromatic residues. The selectivity of FTase for the shorter FPP (5 carbon) substrate is explained by the presence of bulky tryptophan and tyrosine residues of the β subunit (W102 and Y365), within the FPP binding pocket. This blocks the binding of the larger 20-carbon GGPP prenyl moiety (47).

To identify the prenyltransferase enzyme and target protein interactions responsible for substrate selectivity, a series of peptide substrates were co-crystallized with each prenyltransferase (26). This study revealed that the various CAAX sequences bind and interact along one face of the funnel-shaped peptide-binding site in both prenyltransferases except for the C-terminal X residue. FTase utilizes two complementary binding pockets for the X residue depending on the identity of that residue. For example, an X residue of S, Q and M interacts with a binding pocket comprised of Y131α, A98β, S99β, W102β, H149β, A151β and P152β, while F, L, H and N residues interact with a separate binding pocket comprised of residues L96, S99, W102, W106, A151 of the β subunit (26). GGTase-I differs greatly in its X residue specificity in that it is composed of one binding pocket which contains the residues T49, H121, A123, F174 of its β subunit. GGTase-I appears to prefer hydrophobic residues at the X position in its target substrates (26). Taken together, differential binding of prenyltransferases for the X residue in the CAAX domain, and the presence or absence of bulky residues in the isoprene-binding site of FTase and GGTase-I underlie the difference between protein farnesylation and geranylgeranylation.

## 2 Protein prenylation in the neural retina

The discovery of farnesylation of rod transducin gamma subunit in the early 1990s (49) spurred a series of protein biochemistry and pharmacological studies investigating protein prenylation in the retina, especially photoreceptor (PR) cells. Investigations of protein prenylation-associated retinopathies (discussed in sections below) have also provided critical insights into the retinal functions of prenylated proteins. We first discuss some important pharmacological and protein engineering approaches that have provided general insights into the role and requirement of protein prenylation in the neural retina.

Several upstream inhibitors of mevalonate pathway are known to suppress protein prenylation by depleting the cellular isoprene pool. The best-known class of drugs that impact protein prenylation are statins. Statins block HMG-CoA reductase activity, the rate limiting step thus decreasing the pathway flux, and thereby decreasing the levels of intermediate isoprenes. Statins mimic the natural substrate molecule, HMG-CoA, and competitively inhibit HMG-CoA reductase (50). Statins therefore inhibit the synthesis of a broad range of important isoprenoid metabolites. Intravitreal injection of lovastatin led to early changes in the structural organization of the neural retina characterized by formation of rosette-like arrangements of PRs and eventually necrosis of the retina by 4 days (51, 52). Surprisingly, pharmacological inhibition of downstream squalene epoxidase in the neural retina, by intravitreal injection of NB-598 did not lead to acute retinal dystrophy despite cholesterol synthesis inhibition (51, 52). Retinal sensitivity to intravitreal injection of lovastatin was found to be due to defective protein prenylation in the retina, suggesting an indispensable role of protein prenylation in retinal development (51, 52).

**Figure 2:**
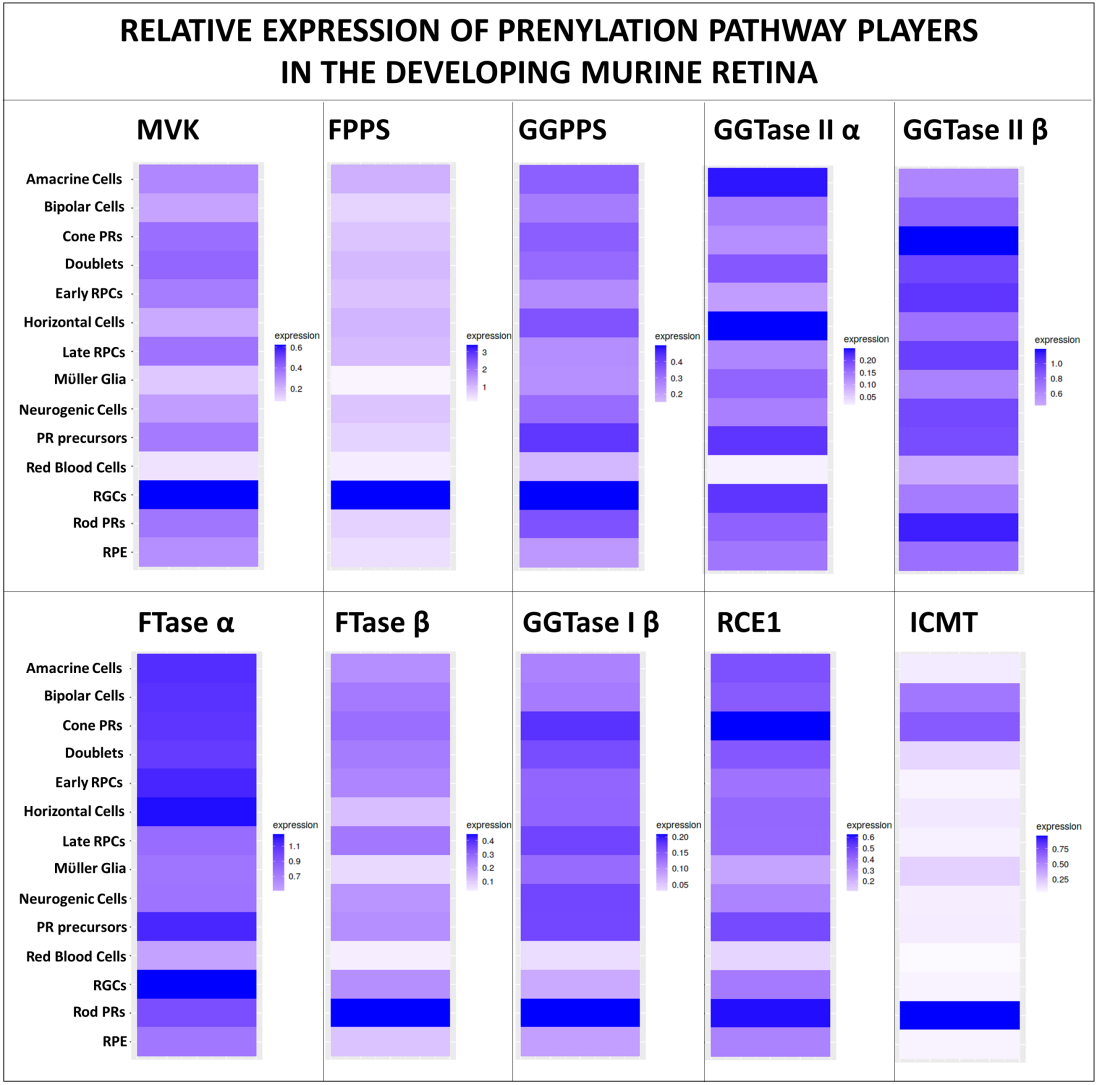
Expression of protein prenylation players in the developing retina. Prenylation is necessary for the maintenance of all retinal cell types, and hence the maintenance of normal retinal structure and function. Query of publicly available mouse retinal single-cell RNAseq dataset (41) using a simple, user-friendly, open source RNA-seq data mining platform (42) demonstrates the expression of key mevalonate pathway players, prenyltransferase subunits, proteases, methyltransferase in essentially all cell types of the developing murine retina (between E11 to PN 8 days).

Other small molecule inhibitors of mevalonate pathway targeting farnesyl diphosphate synthase (FPPS) and geranylgeranyl diphosphate synthase (GGPPS) like the nitrogen-containing bisphosphonates (N-BPs) and their analogues have been studied in the setting of lytic bone disease and cancer (53, 54). Prenyltransferases have been extensively targeted for developing cancer therapeutic compounds such as tipifarnib (FTase inhibitor) and GGTI-2418 (GGTase-I inhibitor) (55–57). The effect of such inhibitors in the developing and mature retina has not been studied, and may provide key insights into the function of prenylated proteins in retinal development and/or retinal pathologies. Post-prenylation enzymes RCE1P, STE24, and ICMT inhibitors have also been of interest as cancer therapeutic strategies (58), but their effects on the retinal structure and function remains to be investigated. Conditional gene deletion strategies or small molecule inhibitor studies are necessary to further elucidate the role and requirement of FTase, GGTase-I, FPPS, GGPPS, and post-prenylation processing enzymes in the maintenance of normal retinal structure and function. Given that protein prenylation is essential in all tissues and cell types, including those in the retina, one may expect pan-retinal expression of critical players of protein farnesylation and geranylgeranylation. We used publicly available datasets and transcriptome datamining tools to demonstrate the expression of key players of protein prenylation pathway in essentially all retinal cell types of the developing murine retina (41, 42) (**Figure 2**).

The mechanism behind the distribution of prenylated targets in rod PRs was explored by Maza and coworkers using chimeric fluorescent probes consisting of different prenyl groups (farnesyl vs. geranylgeranyl), and charged or neutral amino acids upstream of the prenylation site (59). It was shown that prenylated fluorescent protein probes exhibit weak membrane binding and are dynamic in their distribution, suggesting that the membrane compartmentalization mechanisms are not dependent on the nature of the lipid moiety alone (59). Endogenously prenylated proteins may be targeted more efficiently to the cell membrane by interacting with dedicated chaperones. The connecting cilium is suggested to act as an active sorting platform, facilitating the interaction of the target prenylated protein with prenyl-binding chaperones like PrBPδ, for efficient trafficking to the rod outer segment (59). Positively charged prenylated proteins were enriched in the synapse and inner segment (IS) compared to their negatively charged counterparts (59). The absence of positively charged prenylated probes from the outer segment (OS) was initially attributed to the stronger binding at nascent and basal discs at the OS-IS junction. However, this behavior was found to be rather dynamic as photobleaching resulted in the decline of signal strength at the OS-IS junction suggesting a nuanced mechanism of compartmentalization that balances specific interactions with dynamic redistribution (59). Lastly, it was shown that PrBPδ, a chaperone for trafficking prenylated proteins to the outer segment, is dependent on specific structural elements beyond just the prenylated site (59). In summary, trafficking of prenylated proteins in PRs is dependent on prenyl-lipid moiety, charge on the upstream amino acids, prenylated cargo interaction with appropriate chaperones for efficient membrane targeting and may be even influenced by light exposure.

## 3 Protein-prenylation associated inherited retinopathies

We have discussed the biochemical basis of protein prenylation, the cellular requirement of *de novo* synthesis of isoprenes, and provided some insights into protein prenylation in the retina. An excellent, comprehensive review by Roosing et. al., covered this topic of interest nearly a decade ago (5). We herein provide the background and updates on protein prenylation-associated inherited retinal diseases, and insights from transgenic mouse models of retinal prenylopathies.

### 3.1 Choroideremia (OMIM #303100)

The first-described example of prenylation defect-induced visual system dysfunction was X-linked choroideremia, wherein point deletion mutation in the gene *CHM/REP1* (Chromosome X, location: Xq21.2), encoding Rab escort protein 1 (REP-1) was identified. REP-1 serves as a chaperone for several Rab proteins and is a crucial accessory protein for GGTase-II. Mutations in *REP1* causes progressive mid-peripheral atrophy of the retina, RPE and the choroid, resulting in progressive vision loss, ultimately leading to blindness (60). More specifically, male patients initially present with night blindness in childhood, which then advances to peripheral visual field impairment and ultimately to total blindness later in life (61). Whereas female carriers are usually asymptomatic but may exhibit a unique speckling pattern on fundus autofluorescence imaging, and sometimes night blindness as well (62).

**Figure 3:**
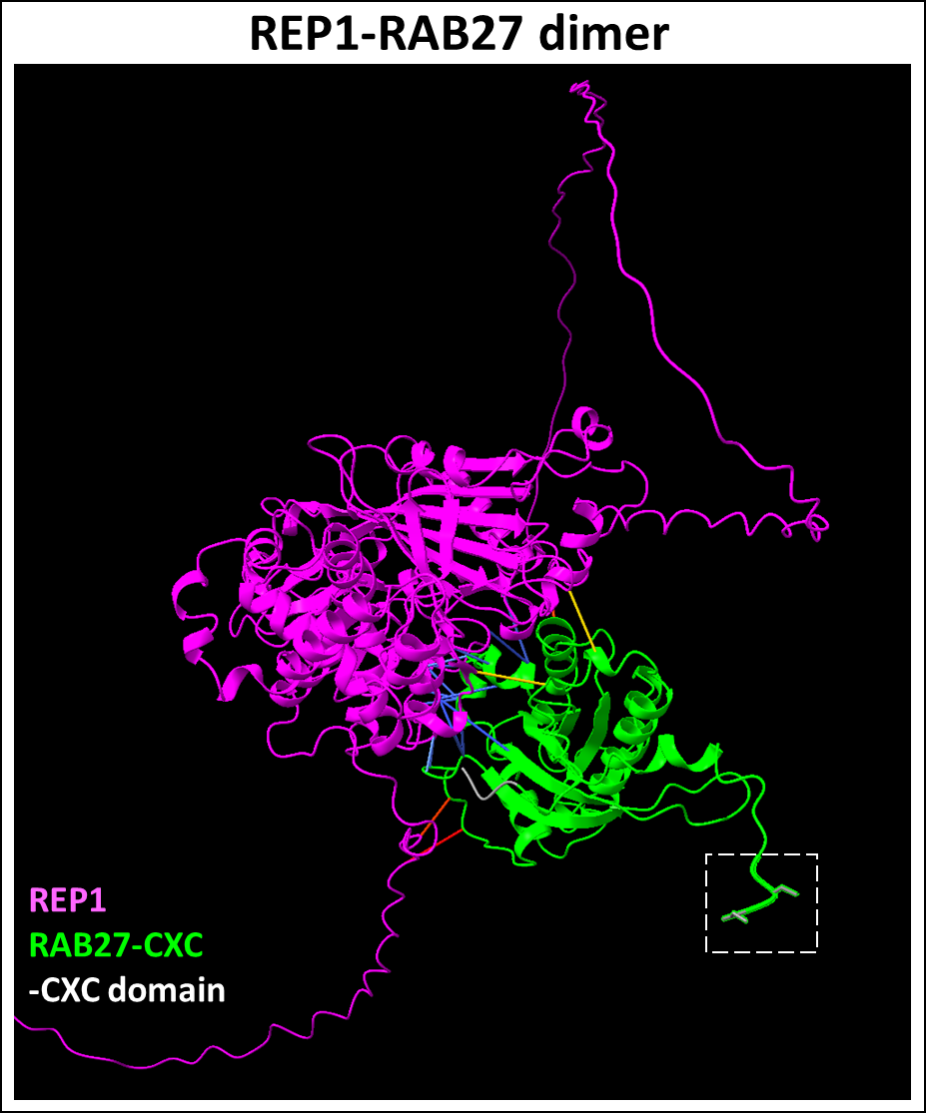
Predicted REP1-RAB27 dimer complex. REP1 (Uniprot ID: P24386) is a chaperone required for presenting Rab27 (Uniprot ID: P51159) to GGTase-II for prenylation at the CXC domain. REP1-Rab27 protein-protein interactions (highlighted in blue) represent predicted binding contacts. Loss of this interaction inhibits the prenylation of the target cargo. Figure generated using ChimeraX (68).

REP-1 chaperones unprenylated Rabs and presents the cargo to GGTase-II for geranylgeranylation (63, 64). REP-1 deficiency leads to the accumulation of unprenylated Rab proteins in the retina (65). Immunolabeling studies showed that REP-1 is expressed in both rods and cones, predominantly in the inner segment and the perinuclear cytoplasm (66). Interestingly, while *CHM* gene expression is found in extraocular tissues as well, the gene mutation predominantly affects the retina and choroid in patients. This is due to shared functional redundancies between REP-2, which may partially compensate for the geranylgeranylation of Rab GTPases in extraocular tissues (67). For example, it was shown that while Rab27a exhibits a similar affinity for REP-1 and REP-2, REP-1-Rab27a complex (**Figure 3**) has a greater preference for GGTase-II compared to the REP-2-Rab27a complex in the retina (69).

The notion of CHM manifesting as a non-syndromic retinopathy has been challenged recently (70). Whole metabolomic analysis of plasma samples from 25 CHM patients versus age- and sex-matched controls showed plasma alterations in oxidative stress-related metabolites, in addition to alterations in tryptophan metabolism, leading to significantly elevated serotonin levels (70). Lipid metabolism was found to be disrupted in CHM patients with evidence of dysfunctional lipid oxidation, and dyshomeostasis in several sphingolipids and glycerophospholipid levels (70). Aberrations in sphingolipid metabolism has been implicated in neurodegenerative diseases, metabolic disorders, immune function, and cancer (71).

Several preclinical and clinical studies targeting CHM have yielded important insights into factors that affect developing effective therapeutic interventions (72–78). *In vivo* studies on *CHM* gene transfer utilizing HIV-based lentiviral vectors led to a decrease in the geranylgeranylated Rab protein load. However, this approach also showed limited transfection of PRs by lentiviral vectors (73). Alternatively, adeno-associated virus (AAV) vectors, such as adeno-associated virus serotype 2 (AAV2) mediated delivery of *CHM* have been shown to achieve efficient transduction of *REP1* in PRs and RPE, with an acceptable safety profile (72, 79–81). However, recent results from phase III of the Efficacy and Safety of BIIB111 for the Treatment of Choroideremia (STAR) trial did not meet the primary endpoint of best-corrected visual acuity (BCVA) for FDA approval. The work highlights the need to consider the stage of disease, anatomical differences, surgical variability, and dose administration among participants (76). Gene therapy for choroideremia may still provide significant visual acuity gains if treated early. Episomal scaffold/matrix attachment region (S/MAR)-based plasmid vectors carrying the human *CHM* gene in CHM patient-derived fibroblasts and a CHM mutant zebrafish model showed some promise. This may provide an alternative delivery strategy for gene augmentation in CHM patients (82).

### 3.2 Phosphodiesterase 6 subunits and its interactors

#### 3.2.1 Phosphodiesterase 6 subunits (OMIM #613801)

**Figure 4:**
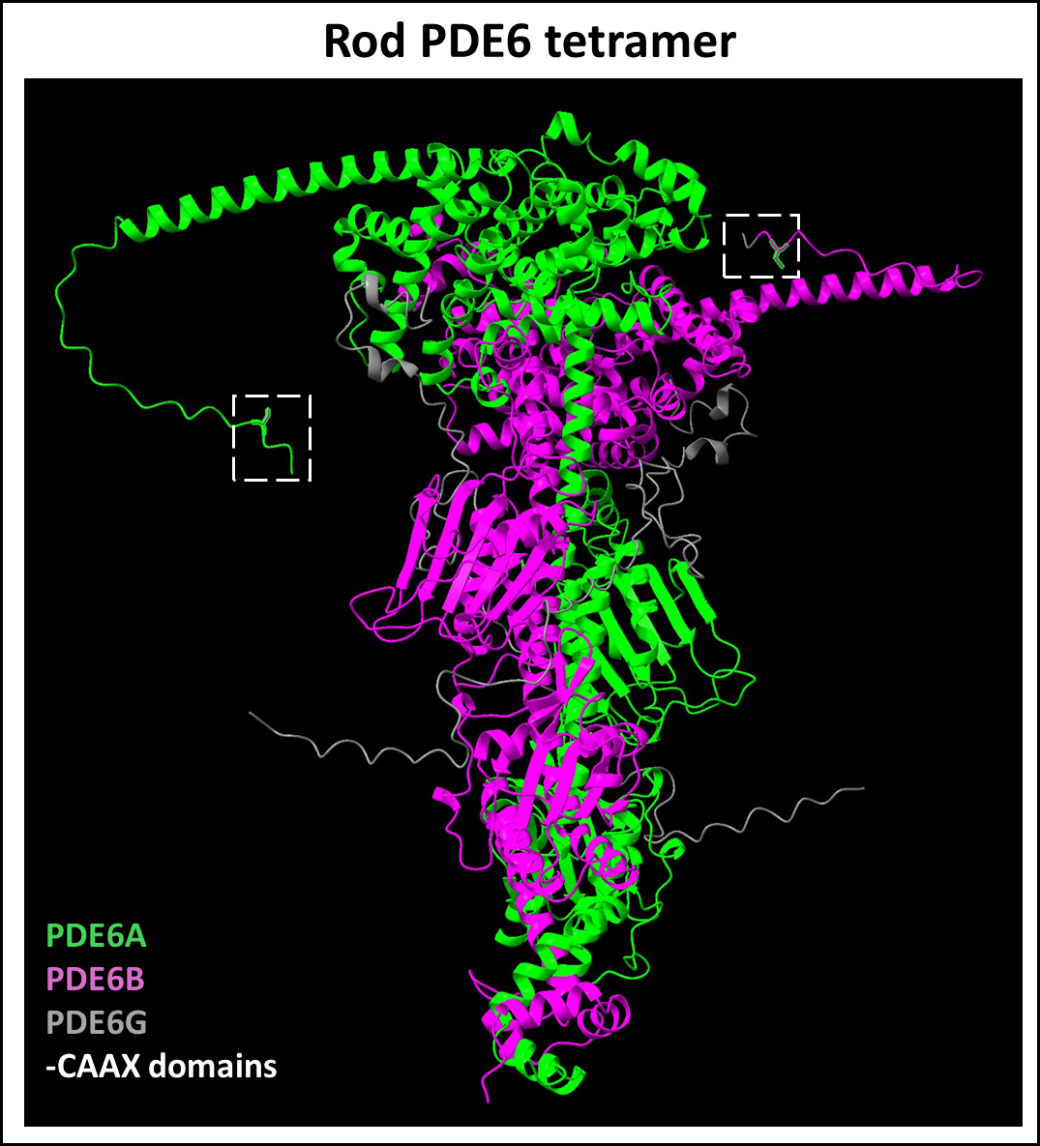
Predicted Rod PDE6 tetramer complex. The rod PDE6 complex is composed of a catalytic PDE6A (Uniprot ID: P16499) and PDE6B (Uniprot ID: P35913) heterodimer with their respective C-terminal prenylation motifs and two copies of small inhibitory γ-subunits (Uniprot ID: P18545). Figure generated using ChimeraX (68).

Proteins of the sixth family of type I cyclic nucleotide phosphodiesterases (PDE6) are key effector enzymes in the phototransduction cascade in rod and cone PRs (83, 84). PDE6 isoforms are regulated by small inhibitory γ-subunits (Pγ); G-protein mediated disinhibition is vital for PDE6 activity and the phototransduction cascade (84). Rod PDE6 complex is composed of a catalytic PDE6A/B heterodimer and two copies of PDE6G (or Pγr) (**Figure 4**), whereas cone PDE6 is a homodimer of catalytic PDE6C subunits each associated with the cone specific Pγc subunit.

**Figure 5:**
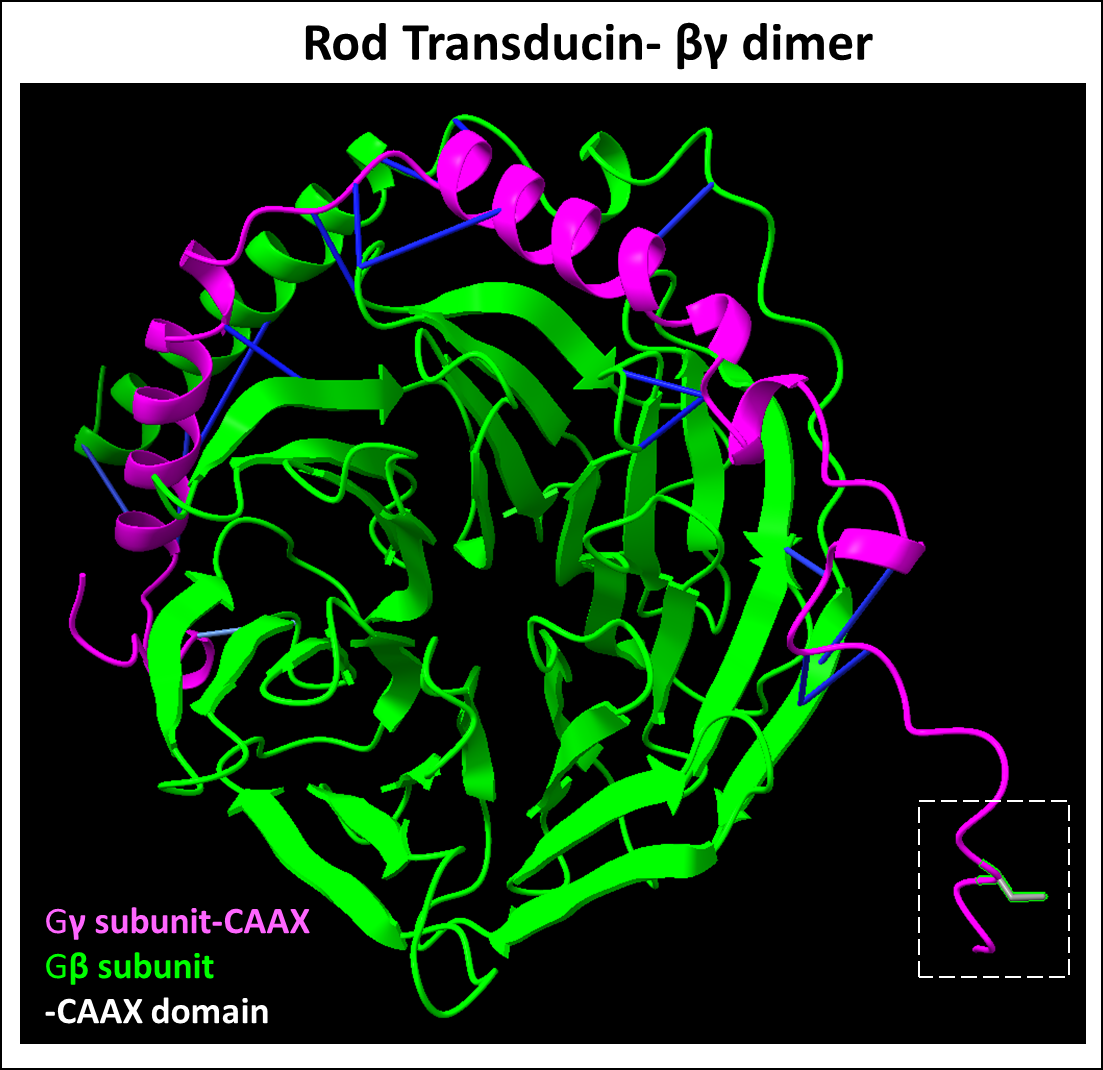
Predicted Rod transducin βγ dimer complex. Transducin βγ dimer is essential for phototransduction. The β (Uniprot ID: P62873) and γ (Uniprot ID: P63211) subunit protein-protein interactions (highlighted in blue) represent predicted binding contacts. The prenylated and processed CAAX motif on the γ subunit serves to enhance membrane binding. Figure generated using ChimeraX (68).

PDE6 complex assembly occurs in the inner segment of PRs including complex formation with HSP90 and AIPL1 co-chaperones. Moreover, the Pγ subunit of transducin also plays a critical chaperone-like role in PDE6 complex formation and was found to cause rapid retinal degeneration in Pγ-deficient knockout mouse model (85, 86). The prenylated and processed CAAX motif on the γ subunit serves to enhance membrane binding of the transducin- βγ dimer (**Figure 5**). It was shown that in contrast to the cone PDE6, the rod PDE6A/B requires Pγ for proper folding and maturation (87). While rod PDE6A is farnesylated, the rod PDE6B subunit and cone PDE6 undergo geranylgeranylation (88, 89). Prenylation facilitates membrane attachment of PDE6 complex and enhances its binding with AIPL1 (90, 91). Rod PDE6A/B not only requires prenylation for membrane trafficking to the outer segment of the retina, but it is also dependent on post-processing by RCE1 (92). Loss of carboxymethylation following prenylation and proteolytic processing does not hinder trafficking of PDE6 complex to the outer segments, but still leads to PR degeneration in an *Icmt* homozygous knockout mouse model (93). Mutations in rod PDE6 are responsible for a small subset of autosomal-recessive RP cases (OMIM #613801) (94, 95). A recent animal model of autosomal recessive achromatopsia (OMIM #613093) highlighted the critical role of prenylation of the catalytic subunit of cone PDE6 (96). Notably, this study showed that cones from homozygous mutants exhibited functional deficits, whereas heterozygous mutant cones were functional. In addition, cones from homozygous mutants displayed structural deficits suggesting a potential role for PDE6 in maintaining cone OS length and morphology (96).

#### 3.2.2 Prenyl binding protein δ (OMIM #615665)

**Figure 6:**
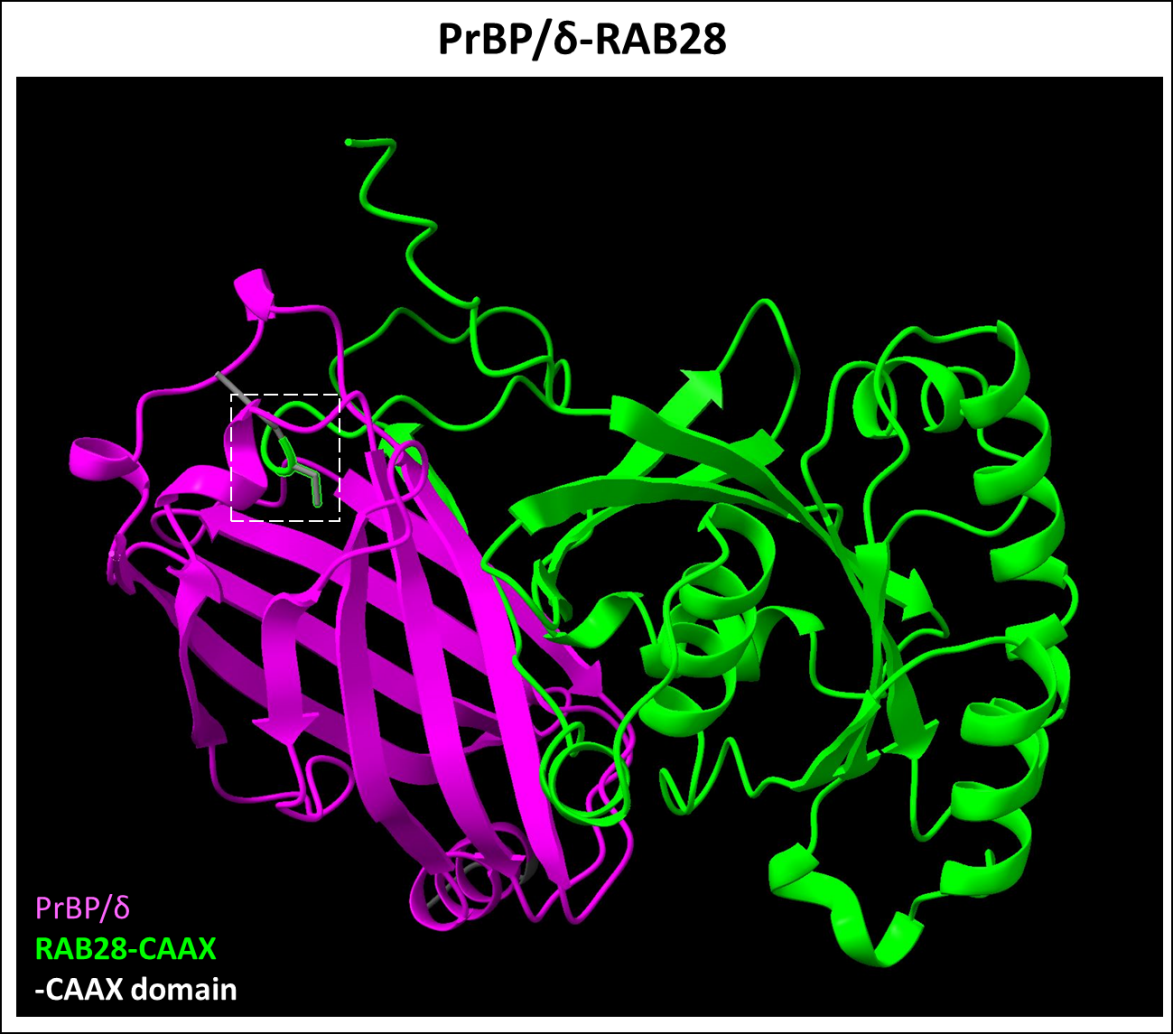
Predicted PrBP/δ-RAB28 dimer complex. PrBP/δ (Uniprot ID: O43924) was recently identified as a Rab28 (Uniprot ID: P51157) protein interactor, which is farnesylation dependent. This interaction allows for the trafficking of Rab28 protein. Figure generated using ChimeraX (68).

**Figure 7:**
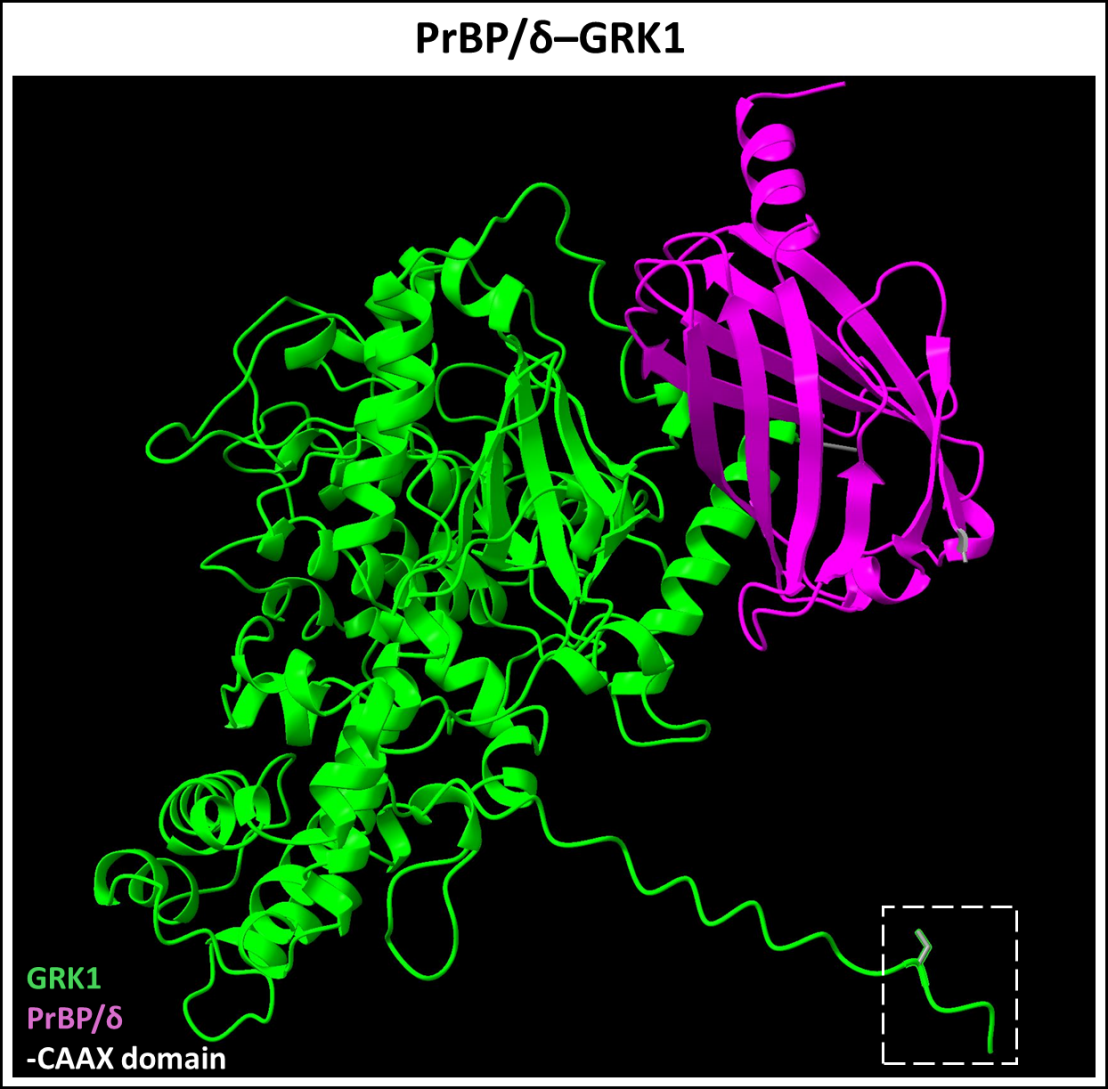
Predicted PrBP/δ-GRK1 dimer complex. PrBP/δ (Uniprot ID: O43924) binds prenylated rhodopsin kinase (GRK1, Uniprot ID: Q15835). This interaction allows for the trafficking of prenylated GRK1 from the inner segment to the outer segment of neural retina. Figure generated using ChimeraX (68).

Prenyl-binding protein (PrBP/δ) is a 17 kDa ubiquitous solubilization factor involved in intracellular trafficking of both farnesylated and geranylgeranylated proteins (97–99). It was first discovered in PR cells as the fourth subunit of cGMP phosphodiesterase 6 (PDE6), hence also termed PDE6δ (97). The crystal structure of PrBP/δ unveiled a hydrophobic pocket between two beta sheets, facilitating the insertion of prenyl groups (100). As an illustration, we have provided the predicted dimerization of PrBP/δ and Rab28 highlighting the hydrophobic interactions between the two proteins (**Figure 6**). In PR cells, PrBP/δ traffics prenylated cargo such as PDE6, Rab28, and rhodopsin kinase (GRK1) from the site of protein synthesis (inner segment) to the retinal outer segment, and its homozygous deletion causes slow, progressive rod-cone dystrophy (101, 102). AlphaFold2/ChimeraX simulation of PrBP/δ-GRK1 complex is provided in **Figure 7**. PrBP/δ was also reported to interact with the prenylated RPGR (retinitis pigmentosa GTPase regulator) isoform, RPGR^1-19^ (103, 104). Mutations in *PDE6D* are associated with Joubert Syndrome (JBTS) (OMIM #615665), a complex neuronal ciliopathy characterized by developmental cerebellar malformations and accompanying retinal degeneration (105, 106).

Several groups showed that PrBP/δ function enhances ciliary targeting of INPP5E, a farnesylated protein implicated in JBTS (OMIM #213300) and Intellectual disability-truncal obesity-retinal dystrophy-micropenis syndrome (OMIM #610156) (105, 107, 108). It is important to note, however, that while the interaction between PrBP/δ and INPP5E is farnesylation dependent, the ciliary targeting of INPP5E is complex and relies on the interplay of four other ciliary localization signals (109). Recently, an affinity proteomics study identified a novel set of PrBP/δ-interacting prenylated proteins that are involved in PR integrity, GTPase activity, nuclear import, or ubiquitination (110). The prenylated protein interactors identified include ciliogenesis factors such as FAM219A, serine/threonine-protein kinase NEM1 (NIM1K), and ubiquitin-like protein 3 (UBL3) (110).

### 3.3 Aryl-hydrocarbon-interacting protein-like 1 (AIPL1) (OMIM #604393)

Several frameshift, missense, and nonsense mutations in the gene coding for Aryl hydrocarbon-interacting protein-like 1 (*AIPL1*) are reportedly associated with autosomal recessive Leber congenital amaurosis type IV (LCA4) (OMIM #604393) (111, 112). Patients with LCA4 typically present with early-onset severe visual impairment, nystagmus, and poor pupillary light responses compared to other LCA types (113, 114). Both scotopic and photopic Electroretinography (ERG) responses were severely diminished in rods and cones, respectively. Disc drusen, optic disc edema, and macular atrophy have also been noted (113, 114). Optical coherence tomography (OCT) imaging in older patients showed severe cone dystrophy (114).

AIPL1 is a specialized PR-specific co-chaperone required for PDE6 maturation and stabilization (115–117). Retinal degeneration caused by AIPL1 defects were described in two mouse models of LCA4 (115, 116). In *Aipl1* knockout mouse model, rapid rod-cone dystrophy was observed by postnatal 3 weeks of age (115). While the *AIPL1 h/h* mouse model, carrying homozygous, hypomorphic mutant allele for *AIPL1*, causes diminished expression of AIPL1, resulting in relatively slower PR dystrophy beginning after 12 weeks of age with almost complete loss of PRs by 5 months of age (116). In both models, PDE6 expression was found to be drastically perturbed, suggesting an indispensable role of AIPL1 in PDE6 maturation.

The AIPL1 protein contains an N-terminal FK506-binding protein (FKBP) domain and a C-terminal tetratricopeptide repeat (TPR) domain with three tetratricopeptide repeats (111). Structural studies confirmed that the PDE6 prenylation is required for its binding to the FKBP domain of AIPL1 (91, 118). The TPR domain serves as a binding site for cytosolic HSP90 and the regulatory Pγ subunit of the PDE6 complex (119, 120). Interaction of PDE6 with the TPR domain of AIP1L also plays a key role in the chaperone-dependent folding and maturation of the PDE6 complex (117, 121). Intriguingly, AIPL1 appears to be colocalized predominantly from the synapse to the inner segment of PR cells, with an enrichment in the connecting cilium, while the PDE6 complex is eventually trafficked to the outer segment (122). This may suggest that AIPL1 chaperone function is limited to PDE6 maturation and does not extend to the trafficking of PDE6 to the outer segments. A recent transgenic mouse study showed that targeted mutation of PDE6A at the prenylation consensus cysteine target, does not affect its trafficking to the outer segment (123). Prenylation of PDE6C was found to be important for the formation of a stable ternary chaperone complex comprising of AIPL1, HSP90, and PDE6 subunits in HEK293 cells (123). Future work is required to determine if AIPL1 plays a role, if any, in a relay-type system where it offloads mature PDE6 complex to another trafficking chaperone like PrBP/δ.

### 3.4 Guanine nucleotide-binding protein subunit gamma, Tγ

Heterotrimeric G-protein transducin, Gt, is an essential signal transducer and amplifier in retinal PR cells for phototransduction (124). It is a membrane-bound G-protein comprised of α, β, and γ subunits, and is bound to a guanine diphosphate (GDP) molecule (124). Both α and γ subunits are lipid-modified, wherein the α subunit is acylated and the γ subunit is farnesylated, despite both subunits possessing a CAAX motif (125, 126). These modifications allow for outer segment membrane localization and subsequent protein interaction (127). Prenylation also enhances the binding of Gβγ to G-protein α subunits (128). The importance of Gγ subunit farnesylation was shown by Kassai and co-workers, who demonstrated that replacing the farnesyl group of Gγ with the more hydrophobic geranylgeranyl attachment rendered the Gβγ dimer incapable of undergoing light-driven translocation to rod outer segments (129).

Homozygous deletion of Gγ subunit (coded by the gene *Gngt1*) in mice showed rod atrophy starting at PN 1 month, with almost complete rod degeneration by 6 months of age and a significant decrease in the protein levels of Gα and Gβ, despite mRNA levels comparable to wild-type controls (130). In contrast, another study found only poor phototransduction signal amplification in intact rods and showed a decline in visual sensitivity of rods in a Gγ-deficient mouse model, without prompt retinal degeneration (131). The pivotal role of farnesylation of Gγ in mediating phototransduction was further confirmed in point mutant models where the Gγ farnesylation site was removed (132). This study showed that farnesylation was not essential for Gβγ dimerization (**Figure 5**), but essential for localization to the rod outer segments and participation in phototransduction (132). Surprisingly, despite the clear functional role of farnesylated Gγ in the phototransduction cascade, there are no known mutations of *Gngt1* linked with inherited retinopathies. This is perhaps attributable to the low frequency of mutation reported in this relatively small gene (225 bp open-reading frame) (5).

### 3.5 RAB28 (OMIM #615374)

Rab small GTPases, which belong to the Ras superfamily, are key regulators of membrane trafficking and cell growth (133, 134). Rab28 carries a C-terminal CAAX motif that is farnesylated in contrast to the common Rab geranylgeranylation and was the first prenylated small Rab GTPase identified to be directly involved in an inherited retinal disease (135–137). Immunohistochemical analysis suggested that Rab28 colocalized at the PR basal body and the ciliary rootlet (137). *Rab28* mutations cause cone-rod dystrophy 18 (CRD18) (OMIM #615374), presenting with central retinal atrophy and hyperpigmented appearance of the fovea. Both scotopic and photopic ERG responses were significantly diminished, suggesting decreased rod and cone response to light, respectively (136–138). A transgenic knockout mouse model of *Rab28* showed Rab28 requirement for disc shedding by cone PRs and its subsequent phagocytosis. Loss of Rab28 function in knockout mice faithfully mimics the human CRD18 phenotype (139). Moreover, this study identified Rab28 protein interactors such as KCNJ13 and PrBP/δ (PRBP/δ and Rab28 complex, simulated in **Figure 6**). Autosomal recessive mutations of *KCNJ13* leads to Leber congenital amaurosis type 16 (LCA16) (OMIM #614186), wherein KCNJ13 regulates phagocytic uptake of shed outer segments by the RPE (139, 140).

### 3.6 Retinitis pigmentosa GTPase regulator (RPGR) (OMIM #300455)

Autosomal dominant mutations in *RPGR* gene (Chromosome X, Xp11.4) account for over 70% of X-linked retinitis pigmentosa cases (XLRP) (OMIM #300455) worldwide (141). Two major spliced isoforms of RPGR have been identified in the mammalian retina: RPGR^1−19^ and RPGR^ORF15^ (142). Both protein isoforms share an identical N-terminal tandem repeat structure named RCC1-like domain (RLD), while the C-terminal domains are not conserved due to altered mRNA splicing. The C-terminus of RPGR^1−19^ constitutes a target prenylation motif which is geranylgeranylated, whereas RPGR^ORF15^ contains a Glu-Gly-rich low complexity region in the C-terminus (142, 143). The shared N-terminal RLD region may confer some functional redundancy between the two RPGR isoforms, while the differential prenylation of RPGR^1−19^ may govern its differential subcellular colocalization and function. This has been demonstrated *in vitro* using transformed human RPE1 cells wherein the prenylated RPGR^1−19^ isoform localized to the RPE primary cilia, while RPGR^ORF15^ does not traffic to cilia (144, 145). Interestingly, rescue experiments in *RPGR* knockout mice showed that the RPGR^ORF15^ isoform sufficiently rescued PR functioning (146, 147).

A report showed a decrease in RPGR^1−19^ isoform, with concurrent upregulation in the RPGR^ORF15^ isoform throughout eye development (147). Thus, suggesting a duality in the role of the two RPGR isoforms during retinal development vs.. functioning of the mature retina. However, the exact role of prenylated RPGR^1−19^ in early retinal development has not yet been directly established. Recently, Zhang *et.al.*, showed the effects of common missense mutations of the shared RLD on the RPGR protein interaction network, and demonstrated that the RLD domain is indispensable for interactions with PrBP/δ and INPP5E in RPE1 cells (148). Prenylation of RPGR^1−19^ isoform is necessary for its ciliary targeting via PrBP/δ trafficking and that prenylation also enhances the RLD binding region for protein interaction (148). Future work should aim to gain the mechanistic understanding underpinning protein interaction disruption on retinal health and whether prenylation represents a dispensable modification in the setting of XLRP pathogenesis.

### 3.7 Mevalonate kinase deficiency (OMIM #610377)

Mevalonate kinase (MVK) catalyzes the phosphorylation of mevalonic acid to phosphomevalonate. Autosomal recessive mutations in *MVK* causes metabolic disorder with poor genotype-phenotype correlation (149). It encompasses a severe form of the disease, mevalonic aciduria (MEVA) (OMIM #610377), and a milder periodic fever syndrome, hyperimmunoglobulinemia D syndrome (HIDS) (OMIM #260920) (150). HIDS is characterized by regular episodes of high fever and systemic inflammation in the affected pediatric patient population. In contrast, mevalonic aciduria patients present with chronic systemic inflammation, along with neurodevelopmental issues such as cerebellar atrophy, dystonia, and ataxia. MKD patients also present with gastrointestinal complications and retinal dystrophy (151–153). A study in 2013 showed that mutations in *MVK* causes late-onset non-syndromic RP, accompanied by mild MKD symptoms (154). It was thought that the accumulation of mevalonic acid may be responsible for the neuronal degeneration including, PR deterioration, observed in MKD patients (153). Two recent studies have shown that patients with MKD exhibit significant accumulation of unprenylated Rab proteins (155, 156). Therefore, the retinal dystrophy phenotype associated with MKD may arise from decreased *de novo* synthesis of isoprenoid metabolites (dolichol, cholesterol, FPP, and GGPP), as well as cellular insufficiency in prenylation of the target proteome.

### 3.8 Autosomal recessive mutations in NUS1/Nogo B Receptor: a putative protein-prenylation disorder (OMIM #617082)

Recent discoveries of novel prenyltansferases, targets motifs, and non-canonical mechanisms of prenylation, has increased the probability of discovering yet unidentified prenylopathies. Here, we briefly discuss the case of a rare autosomal recessive mutation that primarily affects the cis-prenyltransferase (CPT) complex involved in dolichol synthesis. The cis-prenyltransferase complex is a heterotetrameric complex comprised of the catalytic subunit dehydrodolichyldiphosphate synthase (DHDDS), and its interacting partner NUS1 or Nogo-B receptor (NgBR). The CPT complex catalyzes the serial *cis*-condensation of multiple IPP molecules to FPP, to ultimately generate dolichol, an isoprenoid metabolite which serves as the obligate glycan carrier, and is indispensable for protein glycosylation. We recently discussed the visual disorders pertaining to the dolichol synthesis pathway (157).

**Figure 8:**
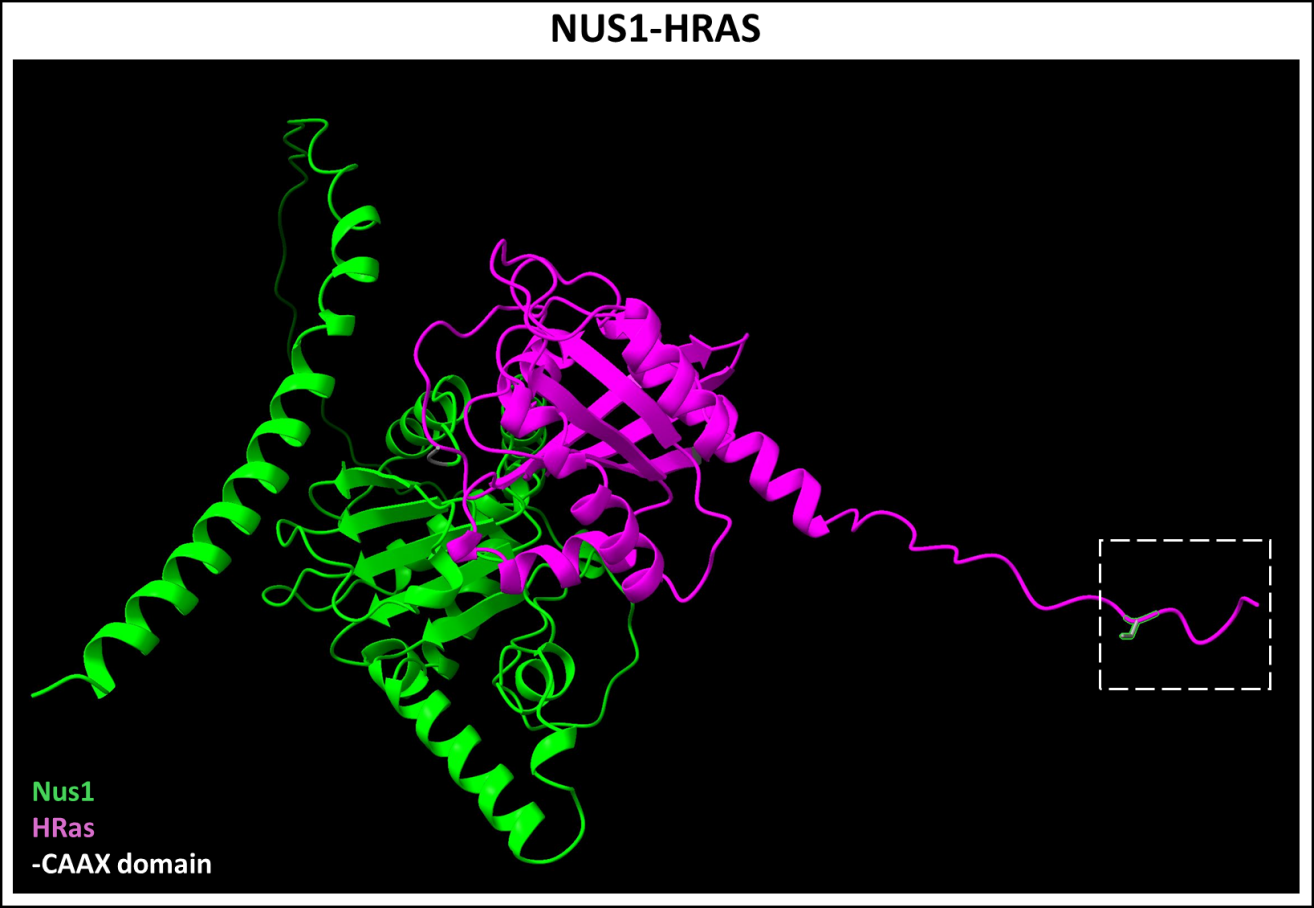
Predicted NUS1-HRAS dimer complex. Nogo B-Receptor (NUS1, Uniprot ID: Q96E22), a part of the cis-prenyltransferase complex, was recently shown to bind farnesylated HRAS (Uniprot ID: P01112) and plays a chaperone-like role in its membrane localization. Figure generated using ChimeraX (68).

Recessive mutations in *NUS1* leads to a systemic disorder including neurodevelopmental issues, retinal dystrophy, and optic neuropathy, unsurprisingly, given the requirement of dolichol synthesis and protein glycosylation in all cells (158). NUS1 performs several other critical roles in maintaining lysosomal homeostasis (159). Interestingly, a recent study showed that NgBR independently binds to farnesylated Ras protein, outside of its CPT function, and plays a key role in membrane localization of H-Ras (**Figure 8**) (160). Moreover, NgBR binds not only to farnesylated H-Ras with its canonical CAAX (CVLS) motif, but also a recombinant geranylgeranylated H-Ras protein with GGTase-I target CAAX sequence (CLVL) (160). We have independently demonstrated that conditional ablation of *Nus1* in the developing PRs, using a CRX-Cre mouse line, causes total PR and bipolar cell loss by PN 2–3 weeks (Ramachandra Rao *et.al.*, unpublished results). However, the underlying degenerative mechanism remains to be understood. These findings highlight the need for investigating the additional chaperone-like roles of NgBR in binding and trafficking prenylated proteins in the retina.

We have provided a brief, tabular summary of the clinical features and molecular basis of known, above discussed prenylation-associated retinopathies in **Table 1**. Defects in prenylation or the chaperone-mediated maturation and trafficking of key membrane-bound prenylated proteins essentially manifest as early rod-cone or cone-rod dystrophies, depending on the predominant affected cell type.

**Table 1:** Overview of ocular outcomes in protein prenylation-associated inherited retinopathies.

DISEASE	RETINAL FINDINGS	OCULAR CLINICAL FEATURES	IMPLICATED PROTEIN	ROLE OF PRENYLATION
**Choroideremia (ref #61) (OMIM #303100)**	Diffuse, progressive degeneration of the retinal pigment epithelium (RPE), photoreceptors and choriocapillaris	Progressive peripheral vision loss, and ultimately central vision loss. Rod-cone dystrophy.	Rab escort protein-1 (REP-1)	Chaperones unprenylated Rab proteins and presents them to GGTase-II for geranylgeranylation
**Retinitis Pigmentosa 40 (ref #94, 95)** (**OMIM #613801)**	Pale retina, attenuated retinal vessels, and typical intraretinal bone spicule pigment in the peripheral retina.	Progressive peripheral vision loss; tunnel vision at the later stages of the disease.	Rod Phosphodiesterase subunits alpha and beta (PDE6α) & (PDE6β)	Prenylation enables proper trafficking of PDE6 from photoreceptor inner segment to the outer segment.
**Achromat-opsia (ref #96) (OMIM #613093)**	Fundus exam can appear normal early in the disease course. Late-stage retinal pigment epithelial mottling and atrophy.	Visual acuity ranges from 20/200 or worse in complete achromatopsia to 20/80 in incomplete achromatopsia. Color vision is severely or completely diminished.	Cone Phosphodiesterase 6 (PDE6C)	Prenylation enables proper trafficking of PDE6C from photoreceptor inner segment to the outer segment.
**Joubert Syndrome (ref# 105, 106)** (**OMIM #615665)**	Early-onset severe rod-cone dystrophy. Late-onset cone-rod dystrophy, and optic nerve atrophy.	Rod-cone dystrophy. Oculomotor abnormalities are common, including oculomotor apraxia, decreased vestibulo-ocular reflex cancellation, compensatory head thrusts or catch-up saccades, and nystagmus.	Prenyl-binding protein (PrBP/δ)	A ubiquitous solubilization factor involved in intracellular trafficking of both farnesylated and geranylgeranylated proteins.
**Leber congenital amaurosis type IV (ref# 113, 114)** (**OMIM #604393)**	Several retinal abnormalities develop in isolation or combination including chorioretinal degeneration and atrophy centered around the fovea, “bone-spicule” like pigmentation, subretinal flecks, pigmented nummular lesions at the level of the RPE, and optic disc abnormalities	Abnormal or absent pupillary response, keratoconus, nystagmus, nyctalopia	Aryl-hydrocarbon-interacting protein-like 1 (AIPL1)	Photoreceptor specific co-chaperone that facilitates PDE6 maturation.
**Cone-rod dystrophy 18 (ref# 136–139)** (**OMIM #615374)**	Macular atrophy and hyperpigmentation. Peripheral cone-rod dystrophy was also noted later in disease course.	Decreased visual acuity and increased photophobia in childhood. Followed by dyschromatopsia, scotomas in the center of the visual field, and partial peripheral vision loss. Progressive peripheral vision loss.	Ras-related protein Rab28	Essential role in cone-specific disc shedding and phagocytosis. Prenylation aids in its localization at the ciliary rootlet.
**X-linked Retinitis Pigmentosa (ref# 142. 147)** (**OMIM #300455)**	Variable depending on the type of photoreceptors affected. Rod predominant dystrophy findings include RPE atrophy, “bone spicule” pigmentary deposits, and retinal vessel attenuation. Whereas cone predominant dystrophy exhibits bullseye maculopathy, eventually developing bone spicule pigmentary deposits and retinal vessel attenuation.	Nyctalopia and peripheral visual field loss are noted early in rod involved dystrophies. Cone dystrophies present with decreased visual acuity, photophobia, and color vision disturbances. Central scotoma is common. With disease progression, cone dystrophies will present with patchy loss of peripheral vision and nyctalopia as rods degenerate.	Retinitis pigmentosa GTPase regulator isoforms (RPGR^1−19^ and RPGR^ORF15^)	Prenylated RPGR^1−19^ is thought to play a role in early retinal development, whereas RPGR^ORF15^ is vital for the maintenance of mature photoreceptors.
**Mevalonate kinase deficiency (ref# 151)** (**OMIM #610377)**	Retinal findings can include RP-type phenotype with RPE atrophy, bone spicule pigmentary deposits, and retinal vessel attenuation.	Uveitis, blue sclera, nyctalopia and peripheral vision loss, and cataracts depending on the form of the disease, ie, MEVA vs. HIDS.	Mevalonate Kinase (MVK)	Essential enzyme in the isoprenoid synthesis pathway. Deficiency leads to lack of FPP and GGPP synthesis, and cellular prenylation defects.

Brief overview of clinical outcomes and molecular basis of known protein prenylation-associated inherited retinopathies. Description of retinal findings and clinical outcomes in known prenylation-associated retinopathies. We have provided appropriate description of the affected gene product, and its putative function in the neural retina.

## 4 Techniques to investigate prenylated proteome

We have thus far discussed the biochemical mechanisms underlying protein prenylation, and inherited blinding diseases characterized by defective prenylation of proteins expressed in the neural retina. With the discovery of novel prenyltransferases and non-canonical target motifs, it would be unsurprising if other prenylation-associated retinopathies and/or systemic disorders are discovered. It is therefore essential to develop approaches to investigate the retinal prenylome. We now turn our attention to technical advances and available methodologies in predicting and/or directly determining the cellular prenylome. We also discuss possible applications of computational, proteomic, and *in vitro* approaches to determine the total retinal prenylated proteome.

### 4.1 Computational approaches to predict cellular prenylome

Computational approaches have been developed to enable prediction of prenylated proteome. Maurer-Stroh and Eisenhaber developed the algorithm PrePS to predict prenyltransferase substrates, based on data from experimentally derived prenylated motifs and prediction of new motifs acting as a substrate for farnesylation or geranylgeranylation (161). The training dataset consisted of 692 FTase and 486 GGTase-I substrates curated by literature survey as well as BLASTP analysis with known prenylated substrates against an NCBI database. In addition, an 11 amino acid sequence upstream of the prenyl cysteine was added to refine the algorithm. With this refinement of PrePS, the algorithm expands the rules which predict the prenylation of a substrate to a 15-amino acid sequence (162, 163). However, experimental determination of the target peptide prenylation is still necessary to determine true false positive rate of this predictive methodology. FlexPepBind is a structure-based computational modeling approach for predicting FTase substrates (162). This algorithm predicts peptide binding through a structure-based modeling approach by aligning different peptide sequences onto a template peptide-receptor complex. This structure-based predictive method was further experimentally validated using *in vitro* assays, wherein 26 of the 29 tested novel peptide were FTase targets suggesting a very low false negative prediction (162).

Computational approaches dependent on training datasets are expected to be biased against non-canonical, unidentified novel prenylation target motifs. To address this discrepancy, a recent study used yeast Hsp40 Ydj1p chaperone as a genetic reporter (164). Ydj1p is prenylated but is subject to the shunt pathway in which the prenylated protein does not undergo the downstream proteolytic processing and remains cytosolic. Prenylated Ydj1p produces a thermotolerant phenotype in yeast which serves as a rapid proxy to monitor protein prenylation status. Using this mutation-phenotype screen, the authors evaluated over 67,000 recombination events, correlating to about 93.5% of the total possible amino acid combinations which may code for CAAX motif. The hits identified from the Ydj1p-based genetic screen did not greatly overlap with those found in other screenings that utilized common targets such as Ras, suggesting that a much larger set of motifs may be serving as prenylation motifs than was previously thought (164). Only a small subset of prenylated motifs identified using the Ydj1p-based screening were identified as having a high probability of prenylation by FlexPepBind and PrePS with results of 27% and 7%, respectively. However, it is important to note that this discrepancy may also reflect different target peptide-specificities for the mammalian and yeast prenyltransferases enzymes.

Data mining methods using experimentally determined retinal proteome and transcriptome data may be highly effective in predicting the retinal prenylome, including non-canonical prenylation motifs. Applying targeted data mining to the transcriptome or proteome specific to the RPE and neural retina—separated into macular and peripheral regions—could be particularly powerful (165). These methods may help distinguish the prenylome specific to the human macula and peripheral retina and allow for better understanding of protein prenylation-associated vision disorders. Furthermore, pathway analysis tools like Ingenuity Pathway Analysis may reveal the potential cellular and retina-specific functions of these predicted prenylated proteins (166). The latest advancements in AI-driven prediction of protein structures, protein-protein interactions, and protein-ligand interactions such as AlphaFold2 and AlphaFold3, could provide preliminary computational validation of these predicted retinal prenylomes (167). It is important to acknowledge that novel hits arising from predictive, computational methodologies need to be experimentally validated using *in vivo* and *in vitro* approaches, as discussed below.

### 4.2 *In vitro* and biochemical approaches for investigating prenylation mechanisms

Fluorescence tagging of proteins has been extensively utilized to study prenylation at endogenous levels, providing insights into prenylated protein maturation and trafficking, and has also facilitated studies of prenyltransferase inhibitors since membrane localization is an easily measurable experimental endpoint. Diffuse fluorescence throughout the cell indicates cytoplasmic localization, which implies a protein either not undergoing prenylation or becoming prenylated without further processing as noted in the shunt pathway. For instance, the effect of proteolysis on membrane colocalization of K-Ras protein was determined using the above discussed chimeric approach (168). Using *Rce1* ^+/+^ and *Rce1* ^-/-^ fibroblast cells transfected with a chimeric GFP-K-Ras construct, it was shown that fluorescence was localized to the plasma membrane in *Rce1* ^+/+^ cells but diffuse in *Rce1* ^-/-^ cells, suggesting that proteolysis was necessary for Ras protein membrane localization (168).

An alternative approach for detecting protein prenylation inside mammalian systems that is not reliant on membrane localization as proxy endpoint, is called Protein Lipidation Quantification (PLQ). This method is derived from an established method of Micellar Electro-Kinetic Chromatography (MEKC) that allows selective partition of prenylated proteins into detergent micelles (using SDS) during capillary electrophoresis (169, 170). Detergent micelles serve as pseudo-stationary phase and exhibit differential electrophoretic migration compared to unprenylated free analyte. PLQ has been successfully used to separate prenylated full length fluorescent protein with a C-terminus CAAX motif in *in-cell* studies (171).

### 4.3 Proteomic and lipidomic approaches for experimental determination of cellular prenylome

Chemical proteomic approaches facilitate the direct detection of prenylation *in vivo* and are based on the development of analogues of FPP and GGPP that are functionalized with biochemical affinity tags, immunogenic tags for detection by antibodies, and chemo-selective tags for bio-orthogonal labeling. Spielmann and coworkers showed the application of an immunogenic tag by using anilinogeraniol (AGOH) to detect FTase substrates (172). Anilinogeraniol is an analogue of an upstream precursor of FPP, which undergoes cellular kinase-dependent diphosphorylation to 8-anilinogeranyl diphosphate (AGPP), and where the third isoprene unit of FPP is replaced with an aniline moiety that then serves as an epitope for detection by Western blot (172, 173). Biotin-geranyl diphosphate (BGPP) has been generated for use as an affinity tag (174). BGPP only allowed for the efficient identification of GGTase-II substrates as the bulky nature of the biotin group was found to interfere with the protein substrate binding for FTase and GGTase-I. Engineered FTase and GGTase-I variants can utilize BGPP as a donor for protein modification (174). Lysates from cells expressing wild-type or the engineered prenyltransferase variants were incubated with BGPP, followed by avidin pull down and proteomic analysis, which allowed the identification of GGTase-II target proteins. Several Rab proteins such as Rab7 and Rab27, which were differentially prenylated in WT prenyltransferase expressing cells, were identified as GGTase-II substrates (174).

Distefano and coworkers designed alkynylated FPP analogue C15AlkOPP, which has been used to study protein prenylation inhibitors, labeling of proteins sensitive to human pathogens, and the delineation of the prenylome of *Plasmodium falciparum*, the causative agent of malaria (175–180). More recently, new alkyne-tagged isoprenoid analogues which closely mimic FPP (YnF) and GGPP (YnGG) allowed click-chemistry based detection of prenylated proteins via mass spectrometry in a human endothelial cell line (181). These analogues allow quantitative analysis of endogenous prenylated proteome since they do not alter the specific activity of prenyltransferases. Upon addition of the alkyne tagged analogue, proteins are captured via click CuAAC ligation to azide-containing fluor/biotin-tagged reagents. This facilitates the enrichment of prenylated proteins and subsequent analysis via LC-MS/MS. These analogues also proved useful in investigating protein prenylation *in vivo.* Intracerebroventricular (ICV) injection of the C15AlkOPP analogue in brains of a transgenic mouse model (APP/PSI) of Alzheimer’s Disease found the upregulation of several prenylated proteins in diseased mice (182). The development of these protein prenylation probes has greatly enhanced our understanding of protein prenylation biochemistry. Appropriate application of similar approaches in *in vitro* and *in vivo* models may prove fruitful in delineating the underlying degenerative mechanisms of prenylation-associated retinopathies.

Experimental determination of retinal prenylome *in vivo* is challenging since current prenylation screening assays utilize *in vitro* approaches. While proteomic and lipidomic strategies have proved to be powerful in refining our understanding of the prenylome, these approaches are expensive and labor-intensive. However, both the *in vitro* as well as the proteomic/lipidomic approaches described above may be highly relevant in defining the retinal cell type-specific prenylome. For instance, RPE prenylome and target-specific prenylation mechanisms may be easily investigated by applying such approaches in induced pluripotent stem cell (iPSC)-derived RPE cells, as well as primary RPE cells (183). Similarly, primary Müller glia and ganglion cell cultures may also be extremely useful in determining retinal cell type-specific prenylome. Identification of novel retinal cell type-specific prenylated hits may then be further validated *in vivo* using careful conditional gene ablation approaches.

## 5 Future directions

**Figure 9:**
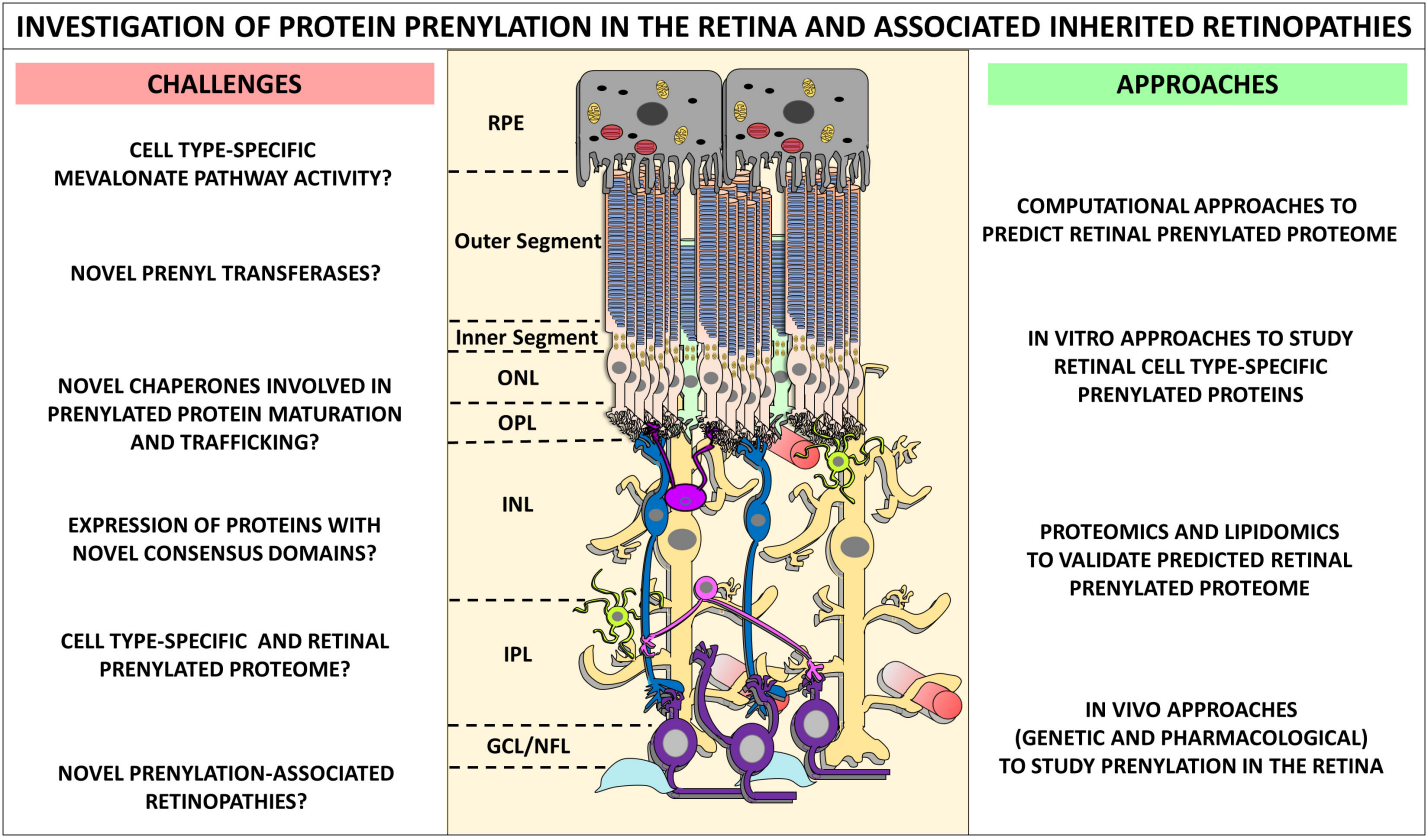
The challenges, approaches and future directions in the investigation of protein prenylation in the retina and associated inherited retinopathies. Determination of retinal prenylated proteome, and the retinal function of such prenylated proteins is challenging. We identify some of the immediate questions and challenges, as well as state-of-the-art approaches in better understanding prenylation mechanisms in the retina. A comprehensive approach linking computational, *in vitro*, proteomics and lipidomics, and *in vivo* strategies can help fully define the retinal prenylome. Such approaches may vastly improve our understanding of prenylation-associated retinopathies, and fast-track the development of therapeutics for these devastating blinding disorders.

Defining the total retinal prenylated proteome, as well as cell type-specific expression, processing, and functions of prenylated proteins will provide a wholesome understanding of the role and requirement of protein prenylation on retinal functioning. Achieving this goal also requires identification and validation of potential novel prenyltransferases, chaperones, and target proteins expressed in the neural retina. As discussed above, careful application of computational approaches will not only facilitate the identification of retinal prenylated proteome, but also help determine differential expression of prenylated proteins in the human macula vs. peripheral retina. These frontiers promise a comprehensive understanding of prenylation-associated retinopathies and their respective mechanisms. The challenges in determining the retinal prenylated proteome, retinal outcomes in inherited prenylopathies, and putative approaches to achieve those ends are summarized in **Figure 9**.

## 6 Concluding remarks

Protein prenylation in PR plays a key role in protein subcellular localization and trafficking. A recent study by Maza *et.al.*, highlights several important considerations and dynamics involved in the subcellular colocalization of prenylated proteins in the PR cell (59). As described, several prenylated retinal proteins are implicated in various retinal pathologies. The underlying role of aberrant prenylated proteins in retinal degeneration may be garnered from their known roles in retinal development and functioning. For example, G protein transducin, PDE6A, and PDE6B are involved in phototransduction cascade, while RAB28 and RPGR are involved in transport regulation. Moreover, mutations affecting AIPL1 and PrBP/δ results in a more generalized prenylation defect as both chaperones are involved in the appropriate trafficking of their cargo prenylated retinal proteins. The reported inherited retinopathies involve the complex interplay of more than just the implicated prenylated protein including protein chaperones and other protein interactors. In the case of MKD, one might hypothesize that supplementing the retina with the deficient isoprenoid groups may rescue the retinal dystrophy phenotype and reverse the accumulation of unprenylated Rabs (5). However, stimulating the synthesis of prenyl groups in the retina may cause undesirable side effects, such as neoplastic growth as is the case with overexpression of prenylated Ras oncogenes products. It is also difficult to predict the impact on isoprene synthesis with this approach given there are multiple feedback mechanisms that regulates mevalonate pathway (18, 184). So far, gene augmentation via lentiviral vectors and more recently, episomal scaffold/matrix attachment region (S/MAR)-based plasmid vectors seem to offer promising avenues in the treatment of prenylation-associated retinopathy such as CHM. However, recent results from phase III STAR trial underscore the need to set realistic expectations for clinical outcomes and emphasize the need for early therapeutic intervention to attain better clinical outcomes.

Finally, to fully capture the role of prenylation in the retina and retinal disorders and thus design therapeutic strategies, it is important to define the complete prenylome of the retina. More than 150 proteins are thought to be involved in the molecular pathways responsible for producing the prenyl lipid moieties or serve as potential substrates for prenylation as determined by PRENbase (http://mendel.imp.ac.at/PrePS/PRENbase/) (44). In addition, it is estimated that over 1166 proteins in the human proteome contain the CAAX motif suggesting that the number of prenylated proteins might be underrepresented in PRENbase. To add to the growing number of potentially prenylated proteins, studies have identified prenylation substrates in both yeast and mammalian systems that diverge from the canonical CAAX box and calls for the expansion of the classically recognized prenylation motif (27, 28). There are estimated to be more than 1000 proteins each with either a C(x)3X or CXX motif at their respective C-terminus. The discovery of heat shock protein chaperone Ydj1p in yeast which undergoes prenylation without subsequent proteolysis or methylation further adds to the complexity of this lipid modification (32). These studies highlight the need to identify potentially “shunted” proteins and proteins containing non-canonical C-terminal sequences, especially in the retina. Application of chemical proteomic approaches, genetic modeling, and targeted mining of retinal proteome to identify putative retinal prenylome will provide a fulsome mechanistic understanding of prenylation-associated blinding disorders.

## Author contributions

SA: Conceptualization, Software, Visualization, Writing – original draft, Writing – review & editing. SR: Conceptualization, Funding acquisition, Software, Supervision, Visualization, Writing – original draft, Writing – review & editing.
